# The criminal gene: the link between MAOA and aggression (REVIEW)

**DOI:** 10.1186/1753-6561-9-S1-A49

**Published:** 2015-01-14

**Authors:** S Sohrabi

**Affiliations:** 1 Medical Student, School of Medicine, University of Glasgow, Scotland, UK

## Background

One emerging aspect of recent advances in neurocriminology is the discovery of possible links between violent criminal behaviour and genetics. Analysis of data from several studies indicates that the strongest link between genetic variation and aggression comes from monoamine oxidase A (MAOA); a gene encoding an enzyme responsible for catabolising amine neurotransmitters such as dopamine, serotonin and noradrenaline. In this work, we present a critical review of the data available from recent investigations regarding the impact of an allelic variation of the MAOA gene on criminal behaviour.

## Methods

The main approach used in this work was reviewing and analysing data presented in a variety of research papers accessed through electronic search.

## Results

The low activity form of the MAOA gene (MAOA-L) has been linked to increased levels of aggression and violence. Data from a 2007 study suggests that MAOA-L individuals are hypersensitive, so are affected more by negative experiences (thus react more aggressively in defence) as opposed to being hyposensitive, and lacking emotion for harming others. Male members of a large Dutch kindred displaying abnormal violent behaviour were found to have low MAO-A activity linked to a deleterious point mutation in the 8th exon of the gene. The unaffected male members within the family did not carry this mutation. The first study that investigated behaviour in response to provocation showed that, overall, MAOA-L individuals showed higher levels of aggression than MAOA-H (high MAOA activity) subjects. There was also strong evidence for a gene-by-environment interaction as both groups showed similar low levels of aggression with low provocation, but MAOA-L individuals displayed significantly higher levels of aggression in a high provocation situation. A further gene-by-environment interaction was found in a long-term study performed on large number of children. Those with the MAOA-L genotype paired with maltreatment in childhood were correctly predicted to commit crime. Similar results are replicated in the majority of other related studies, but not all.

## Conclusions

We present mounting evidence that biological, environmental, and social factors are involved in criminal behaviour. Deficiencies in MAO-A activity have been identified in numerous studies to correlate positively with aggressive behaviour, but its influence may be moderated by environmental factors. Although further research into this aspect of neurocriminology is required, the findings highlight an ethical dilemma with regards to prosecuting criminals. Since individuals cannot be held accountable for their genes, should they be held responsible for their dispositions and resulting actions?

